# European clinical guidelines for Tourette syndrome and other tic disorders—version 2.0. Part III: pharmacological treatment

**DOI:** 10.1007/s00787-021-01899-z

**Published:** 2021-11-10

**Authors:** Veit Roessner, Heike Eichele, Jeremy S. Stern, Liselotte Skov, Renata Rizzo, Nanette Mol Debes, Péter Nagy, Andrea E. Cavanna, Cristiano Termine, Christos Ganos, Alexander Münchau, Natalia Szejko, Danielle Cath, Kirsten R. Müller-Vahl, Cara Verdellen, Andreas Hartmann, Aribert Rothenberger, Pieter J. Hoekstra, Kerstin J. Plessen

**Affiliations:** 1 Department of Child and Adolescent Psychiatry, TU Dresden, Fetscherstrasse 74, 01307 Dresden, Germany; 2 Department of Biological and Medical Psychology, Faculty of Psychology, University of Bergen, Bergen, Norway; 3 Regional Resource Center for Autism, ADHD, Tourette Syndrome and Narcolepsy Western Norway, Division of Psychiatry, Haukeland University Hospital, Bergen, Norway; 4 Department of Neurology, St George’s Hospital, St George’s University of London, London, UK; 5 Paediatric Department, Herlev University Hospital, Herlev, Denmark; 6 Child and Adolescent Neurology and Psychiatry, Department of Clinical and Experimental Medicine, University of Catania, Catania, Italy; 7 Vadaskert Child Psychiatric Hospital and Outpatient Clinic, Budapest, Hungary; 8 Institute of Clinical Sciences, University of Birmingham, Birmingham, UK; 9 Child Neuropsychiatry Unit, Department of Medicine and Surgery, University of Insubria, Varese, Italy; 10 Department of Neurology, Charité Universitätsmedizin Berlin, Berlin, Germany; 11 Institute of Systems Motor Science, University of Lübeck, Lübeck, Germany; 12 Department of Neurology, Medical University of Warsaw, Warsaw, Poland; 13 Department of Bioethics, Medical University of Warsaw, Warsaw, Poland; 14 Division of Neurocritical Care and Emergency Neurology, Department of Neurology, Yale School of Medicine, New Haven, CT USA; 15 Department of Psychiatry, University Medical Center Groningen, Rijks Universiteit Groningen, GGZ Drenthe Mental Health Institution, Assen, The Netherlands; 16 Clinic of Psychiatry, Social Psychiatry and Psychotherapy, Hannover Medical School, Hannover, Germany; 17 PsyQ Nijmegen, Parnassia Group, Nijmegen, The Netherlands; 18 TicXperts, Heteren, The Netherlands; 19 Department of Neurology, Sorbonne Université, Pitié-Salpetriere Hospital, Paris, France; 20 National Reference Center for Tourette Disorder, Pitié Salpetiere Hospital, Paris, France; 21 Clinic for Child and Adolescent Psychiatry and Psychotherapy, University Medical Center Gottingen, Gottingen, Germany; 22 Department of Child and Adolescent Psychiatry, University of Groningen, University Medical Center Groningen, Groningen, Netherlands; 23 Division of Child and Adolescent Psychiatry, Department of Psychiatry, Lausanne University Hospital, University of Lausanne, Lausanne, Switzerland; 24 Child and Adolescent Mental Health Centre, Mental Health Services, Capital Region of Denmark, Copenhagen, Denmark

**Keywords:** Tics, Tourette syndrome, Pharmacotherapy, Medication, Treatment

## Abstract

In 2011, the European Society for the Study of Tourette Syndrome (ESSTS) published the first European guidelines for Tourette Syndrome (TS). We now present an update of the part on pharmacological treatment, based on a review of new literature with special attention to other evidence-based guidelines, meta-analyses, and randomized double-blinded studies. Moreover, our revision took into consideration results of a recent survey on treatment preferences conducted among ESSTS experts. The first preference should be given to psychoeducation and to behavioral approaches, as it strengthens the patients’ self-regulatory control and thus his/her autonomy. Because behavioral approaches are not effective, available, or feasible in all patients, in a substantial number of patients pharmacological treatment is indicated, alone or in combination with behavioral therapy. The largest amount of evidence supports the use of dopamine blocking agents, preferably aripiprazole because of a more favorable profile of adverse events than first- and second-generation antipsychotics. Other agents that can be considered include tiapride, risperidone, and especially in case of co-existing attention deficit hyperactivity disorder (ADHD), clonidine and guanfacine. This view is supported by the results of our survey on medication preference among members of ESSTS, in which aripiprazole was indicated as the drug of first choice both in children and adults. In treatment resistant cases, treatment with agents with either a limited evidence base or risk of extrapyramidal adverse effects might be considered, including pimozide, haloperidol, topiramate, cannabis-based agents, and botulinum toxin injections. Overall, treatment of TS should be individualized, and decisions based on the patient’s needs and preferences, presence of co-existing conditions, latest scientific findings as well as on the physician’s preferences, experience, and local regulatory requirements.

## Introduction

The first European clinical guidelines for Tourette Syndrome (TS^1^) were published in 2011 [1] by working groups of the European Society for the Study of Tourette Syndrome (ESSTS) and provided recommendations for the assessment and treatment of TS based on existing guidelines, meta-analyses, reviews, clinical trials, and case studies up to that point. The present guideline provides clinicians an update of recommendations for the pharmacological treatment of TS in Europe using evidence from clinical trials and clinical expertise.

In general, clinical guidelines rely on the combination of information from controlled clinical trials (including their shortcomings) and clinical (consensus-based) knowledge, given the lack of sufficiently comprehensive and detailed evidence. Regarding TS, the situation mentioned in our 2011 article with “…only a limited number of studies on pharmacological treatment options for TS met rigorous quality criteria…” still holds true. Especially head-to-head comparisons of different agents or their combination as well as optimal treatment duration and dosage have not been systematically investigated, hence calling for an approach supplemented by knowledge from clinical practice. Moreover, the effectiveness of pharmacological treatments in reducing tics varies between trials as a result of differences in methodology and patient characteristics. Furthermore, controlled studies in treatment resistant cases are lacking. It remains common practice to have to try various options until an effective reduction of tics is achieved [2].

Recently, a systematic review and guidelines of the American Academy of Neurology (AAN) for the treatment of TS have been published [3, 4]. The authors of the AAN guidelines used structured, evidence-based methodology as outlined in the 2011 edition of AAN's guideline development process manual. To formulate new European recommendations for the pharmacological treatment of TS, we complemented the English-language literature since 2011 and combined it with the results of a survey among ESSTS experts, who were asked about their pharmacological daily practice in children and adults with TS.

## Methodology of selection of agents and literature search strategy

To select relevant agents, we combined agents with at least moderate or low evidence according to the guidelines of the AAN [3, 4] with those mentioned in our European survey. For these agents, we reviewed the English-language literature since 2011 in PubMed using the agent’s name in combination with “tics”, “tic disorder”, or “Tourette Syndrome”, including children, adolescents, and adults as search string. In addition, we checked the references since 2011 of other systematic reviews/meta-analyses [5–9], existing guidelines [3, 4, 10–12], non-systematic reviews on TS with statements about pharmacological treatment, i.e., dealing with various agents [13–38] or mentioning treatment in their title [39–64]. In addition, we had a look into the references of agent-specific reviews and meta-analyses of (in alphabetical order) aripiprazole [65–72], atypical antipsychotics [73], botulinum toxin [74–85], cannabis [86–88], clonidine [89, 90], complementary alternative medicine [91], deutetrabenazine [92], non-dopaminergic agents [93], traditional Chinese medicine [94], and topiramate [95, 96]. Moreover, we screened references of reviews on specific aspects of TS if they describe treatment options for co-existing attention deficit hyperactivity disorder (ADHD) [97–103], obsessive–compulsive disorder (OCD; [104, 105], autism and stereotypies [106, 107], adverse events of pharmacological treatment in TS [108, 109], and treatment resistant TS [110].

### Agents from recently published AAN guidelines

The authors of the AAN guidelines included only systematic reviews and randomized controlled trials (RCTs) on the treatment of tics that included at least 20 participants. They concluded that there is “….moderate confidence that haloperidol, risperidone, aripiprazole, tiapride, clonidine, botulinum toxin injections, 5-ling granule, and Ningdong granule were probably more likely than placebo to reduce tics….”. Lower confidence was reported for pimozide, ziprasidone, metoclopramide, guanfacine, topiramate, and tetrahydrocannabinol (THC). Strong confidence was demonstrated only for behavioral approaches for tics (for detailed description of the behavioral approaches consult Part II of our guidelines).

### Agents mentioned in the ESSTS survey

In the survey of the ESSTS Guidelines Group conducted in 2019, ESSTS experts’ prescription practices for the treatment of TS were gathered. They were asked which medication they would consider as first, second, third, and subsequent choices, provided absence of contra-indications for the available agents and absence of co-existing conditions. Contrary to our prior survey from 2011 [111], we also asked the experts to give their recommendations separately for children/adolescents and adults.

**Fig. 1:**
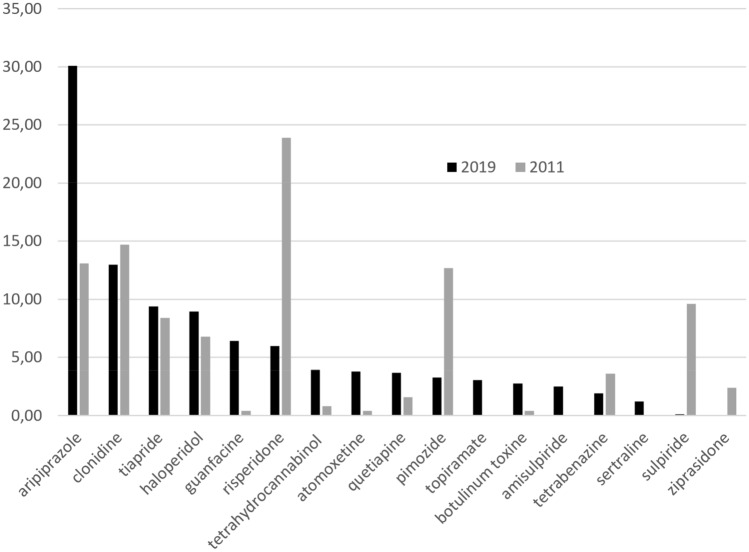
Results from ESSTS surveys on preferences of agents for the treatment of tics in 2011 compared to 2019. In 2011, responses from 22 TS experts were received, while in 2019 50 ESSTS experts (45 responses for treatment in adults and 50 in children/adolescents, findings shown together). Each first choice agent was rated with 4 points, a second-choice agent with 3 points, a third-choice agent with 2 points, and additional agents with 1 point. In 2011, 1 point was given for desipramine, thioridazine, oxcarbazepine (not shown in the figure)

In general within the answers of 59 clinicians, choices in children/adolescents did not differ from those in adults and pointed to a high preference for aripiprazole in both age groups. The main difference between the age groups was that haloperidol was much more commonly considered in adults, while in children/adolescents tiapride was more often mentioned (for details consult, Table 1). When comparing the results of our ESSTS surveys performed in 2011 and in 2019, a clear shift over the last decade can be seen from risperidone, pimozide, and (ami)sulpiride in favor of aripiprazole (Fig. 1).

**Table 1:** Preferences of agents for treatment of TS

Children and adolescents (*n* = 15 different agents were given)			Adults (*n* = 14 different agents were given)		
Points	Percentage		Points	Percentage	
141	29.2	Aripiprazole	127	31.0	Aripiprazole
82	17.0	Clonidine	70	17.1	Haloperidol
81	16.8	Tiapride	37	9.0	Clonidine
49	10.1	Guanfacine	32	7.8	Risperidone
25	5.2	Atomoxetine	26	6.3	Quetiapine
20	4.1	Risperidone	20	4.9	Botulinum toxin
18	3.7	Topiramate	17	4.1	Cannabinoids
18	3.7	Cannabinoids	14	3.4	Pimozide
15	3.1	Pimozide	11	2.7	Guanfacine
11	2.3	Amisulpiride	11	2.7	Amisulpiride
8	1.7	Tetrabenazine	10	2.4	Topiramate
5	1.0	Quetiapine	10	2.4	Atomoxetine
4	0.8	Haloperidol	9	2.2	Tetrabenazine
3	0.6	Botulinum toxin	8	2.0	Tiapride
2	0.4	Sertraline	8	2.0	Sertraline
1	0.2	Sulpiride			
483	100		410	100	

Choices are given separately for children/adolescents and adults. We received 50 responses for children/adolescents and 45 responses for adults (from 50 ESSTS experts; overlap in many cases). We rated each first-choice agent with 4 points, a second-choice agent with 3 points, a third-choice agent with 2 points, and additional agents with 1 point. To enable a comparison of the preferences between both age groups we calculated percentages

We also collected experts’ opinion on the reason for starting pharmacological treatment of TS. Fifty-nine clinicians treating patients with TS who were members of ESSTS (95% from Europe) regarded as most important reason that the “patient/parents requested medication”, followed by “behavior therapy had not been successful”, and finally “high severity of tics”. While 81% of clinicians would use pharmacotherapy as first-line to treat severe tics, in the case of moderate tics this was done only by 34%, and in the case of mild tics by 3% of clinicians (for detailed description of the survey’s results consult Part V of this issue).

### Selection of agents to be discussed in detail

From the moderate confidence group in the AAN guidelines [3] all agents except 5-ling granule and Ningdong granule were commonly prescribed by ESSTS experts (see Table 1). From the lower confidence group only ziprasidone and metoclopramide were not prescribed by ESSTS experts. Vice versa, amisulpiride, tetrabenazine, quetiapine, sertraline, atomoxetine, and sulpiride were mentioned by ESSTS experts as prescribed agents but not in the list of agents with at least moderate or low evidence according to the AAN guidelines.

### Dysfunction of transmitter systems as basis for psychopharmacological treatment

TS has been associated with a dysfunction of many neurotransmitter systems, especially the dopaminergic system [39, 112]. Based on findings from nuclear imaging studies, four hypotheses on dopamine dysfunction in TS have been postulated: (1) dopamine hyper-innervation within the striatum; (2) supersensitive postsynaptic striatal dopamine receptors; (3) presynaptic dopamine abnormality in dopa carboxylase; and (4) elevated intrasynaptic dopamine release as a result of an imbalance between tonic and phasic dopamine levels [112]. Besides a dysfunction in the dopaminergic system, imbalances in other neurotransmitter systems have been suggested including serotonergic, noradrenergic, glutamatergic, GABAergic, cholinergic, histaminergic, endocannabinoid, and opioid systems [34, 39]. These findings give some rationale to use substances other than dopamine blocking drugs particularly for the treatment of tics resistant to dopamine-modulating agents.

### Dopamine-modulating agents

The first agents used in treating tics were first-generation antipsychotics approved by the Food and Drug Administration (FDA), e.g. haloperidol (in 1969) and pimozide (in 1984), whereas aripiprazole was approved in 2014. In European countries, only haloperidol has been licensed for TS [113]. Discrepancies concerning the licensing of the same agents in Europe and the US largely stem from differences in interests of pharmaceutical companies between the US and Europe. In general, adverse effects of dopamine receptor antagonists are broad, ranging from extrapyramidal adverse effects including acute dystonia, parkinsonism, and akathisia to metabolic adverse effects including weight gain, type 2 diabetes mellitus, lipid spectra abnormalities, blood pressure changes, fatigue, headache, body temperature dysregulation, hyperprolactinemia, and sexual dysfunction, increase of prolactin levels, and QTc-prolongation as well as ‘behavioral’ changes, such as concentration problems, apathy, anhedonia and sedation, aggression, anxiety, and agitation [114, 115]. The limited existing evidence does not allow to predict individual potential adverse effects preceding the start of treatment [116]. Regarding hyperprolactinemia, antipsychotics with tight D2-binding predictably lead to hyperprolactinemia (“prolactin-raising”), whereas antipsychotics with loose D2-binding and partial agonistic action (e.g., aripiprazole) are considered as “prolactin-sparing” [117]. While most experts recommend measurement of prolactin levels before and during treatment with antipsychotics, long-term effects of hyperprolactinemia on sexual, bone, and breast development without accompanying acute clinical symptoms are unknown. Accordingly, the question of whether switching to another antipsychotic only because of hyperprolactinemia (without any clinical symptoms) is still a matter of debate [109, 118].

First-, second-, and third-generation antipsychotic agents are all being used in TS. First generation antipsychotics show predominantly extrapyramidal adverse effects and sedation, while second-generation drugs have more metabolic adverse effects (i.e., weight gain, disturbed blood lipid spectra, a higher risk of diabetes, and other metabolic adverse effects [108, 119, 120]). Second- and third-generation antipsychotics are more widely used and have a mode of action that is distinct from first generation antipsychotics’ mode of action, by their binding to both dopamine and 5-HT2 receptors, i.e., their 5-HT2A receptor antagonism [51]. In line with this observation and despite the absence of regulatory approval for their use in TS for the majority of agents (with the exception of aripiprazole in the US), treatment recommendations favor the use of newer generation antipsychotics rather than first generation antipsychotics as first line treatment for tics, predominantly because of a more favorable tolerability with respect to extrapyramidal adverse effects [111, 115, 121]. In the following paragraphs we review the various antipsychotics that may be used as treatment of TS.

### First-generation antipsychotics

#### Haloperidol

Haloperidol was the first antipsychotic medication proven to be effective in the treatment of TS and is in the EU still the only agent with approval for TS. It is a potent dopamine antagonist, specifically against dopamine D2 receptors. It also blocks muscarinic acetylcholine receptors as well as adrenergic receptors and has a well-documented effectiveness in tic reduction [6, 111]. Since 2011, no new English-language RCTs have been published in TS. The most recent meta-analysis (including also Chinese-language RCTs) of haloperidol as treatment for TS pointed to a standardized mean difference compared with placebo of 3.20 (95% CI [0.14–6.52]) [6].

However, due to considerable adverse effects (particularly parkinsonism, apathy, anhedonia, and QTc-prolongation), the use of haloperidol as treatment of TS has decreased within the last three decades from being a first-line agent to being used only in carefully selected, severely affected, and otherwise treatment resistant patients [3, 4, 10].

#### Pimozide

Pimozide is a dopamine D2 receptor antagonist which also blocks calcium channels. In the past, it has been one of the most frequently used medications in the treatment of tics [115] despite only a limited number of trials comparing its effectiveness to placebo or other agents [122]. Since 2011, no new English-language RCTs have been published in TS. Although several reviews give some support that pimozide is effective as treatment of tics, a recent meta-analysis (including also Chinese-language RCTs) did not find that pimozide is significantly better than placebo [6]. Moreover, due to its prominent adverse effects including drowsiness and risk of extrapyramidal symptoms (EPS), although to a lesser extent as compared to haloperidol [9, 122], weight gain (less than risperidone, but more than aripiprazole), sedation [9, 122–124], and the risk of QTc prolongation [9, 122], its application has declined noticeably [125]. Similar to haloperidol, in current guidelines it is recommended only in severely affected and otherwise treatment resistant patients [10, 12].

### Second- and third-generation antipsychotics

#### Risperidone

Risperidone acts through a dopamine D2 receptor and 5-HT2 receptor antagonism [126]. It is one of the best studied antipsychotics for the treatment of tics [16].

Three systematic reviews [8, 9, 90] describing the effect of risperidone on tics have been published since 2011, indicating that risperidone is effective in reducing tics. The most recent meta-analysis (including also Chinese-language RCTs) of risperidone as treatment for TS pointed to a standardized mean difference compared with placebo of 3.47 (95% CI [0.37–6.87]) [6]. One RCT [127] in 60 subjects compared the effects of aripiprazole and risperidone in children and adolescents over a 2-month period, with the conclusion that both medications were tolerated well, with equal effectiveness and similar adverse effects, including increased appetite in over 25% of the participants for either agent. Risperidone, in contrast, was superior in improving the patients' social functioning in the short term.

In terms of adverse effects, 35% of children with tics using risperidone developed EPS in a prospective longitudinal study on antipsychotic safety monitoring [128]. Risperidone-related weight gain seems to follow a pattern with a significant increase of body mass index (BMI) in the first month, followed by only a slow increase thereafter [129, 130]. Increase of prolactin levels was present in 41% of the children treated for tics [128].

#### Aripiprazole

Aripiprazole reduces dopaminergic neurotransmission through D2 partial agonism [131–133]. Thus, it modulates neurotransmission in dopaminergic (mainly mesolimbic and mesocortical) pathways. In addition, it is a partial 5-HT1A agonist and a 5-HT2A antagonist [131–134]. Aripiprazole has become a frequently used agent for the treatment of tics due to its good effectiveness on tics and less prominent adverse effects [50, 69, 72, 73, 111, 135].

Until 2011, the use of aripiprazole was only reported in case studies, retrospective observational studies, and open-label trials [51]. Thereafter, aripiprazole has become the main focus in research on the pharmacological treatment of tics: seven systematic reviews including five meta-analyses or combinations of the two [6, 8, 66, 68, 69, 71, 72] and two placebo controlled RCTs [136, 137] have been published since 2011. All publications consistently documented the effectiveness of aripiprazole in reducing tics, with similar effect sizes as compared to other dopamine-modulating agents, such as haloperidol and risperidone [6, 127, 138]. The most recent meta-analysis (including also Chinese-language RCTs) pointed to a standardized mean difference of aripiprazole compared with placebo of 4.74 (95% CI [1.06–8.67]) [6]. Moreover, there is some evidence from an open-label study that aripiprazole may also have a positive effect on co-existing conditions, such as depression, anxiety, and auto-aggression in adults with TS [135], as well as on social adjustment and parental stress [139]. However, it may have an unfavorable effect on complex learning tasks [140].

Aripiprazole has a more favorable profile of adverse effects compared to other antipsychotics with lower risk of akathisia and other EPS, anxiety, constipation, dizziness, headache, insomnia, nausea, and vomiting in patients with TS [6, 8, 111, 137]. Based on a study in children and adolescents with TS, aripiprazole has a safer cardiovascular profile than pimozide, with a lower frequency of QTc prolongation [125]. Importantly, aripiprazole is less sedating than most other antipsychotics in patients with psychosis [141]. However, more recent literature showed a mean EPS incidence of 17.1% (95% CI 12.8–22.3%) in children and adolescents with a variety of psychiatric disorders treated with aripiprazole [142]. As described in other antipsychotic agents, children gain more weight due to aripiprazole than adolescents and adults [120, 137], but among all antipsychotics aripiprazole has a relatively favorable metabolic profile [143].

### Benzamides

#### Tiapride

Tiapride, a benzamide with low antipsychotic action, acts as a selective dopamine antagonist at dopamine D2 and D3 receptors. Despite its frequent use, particularly in German speaking Europe, the evidence on its effectiveness in the treatment of tics in controlled trials is still sparse [52, 144]. While tiapride is not available in the US [145], in recent years, there was a growing interest in this agent in other countries, such as China [6, 138] and two Chinese guidelines recommended tiapride as first-line medication (Chinese medical association [11]; Chinese Child Neurology Society [12]).

Since 2011, there has been one new English-language RCT [138] and more than 10 Chinese-language RCTs (not cited here, for an overview see [6])—the first ones after the small scale RCT from 1988 [146]. Since 2011, five reviews including one meta-analysis [6, 8, 69, 71, 72] covered also tiapride for the treatment of tics, while others did not even mention tiapride [40, 58]. The most recent network meta-analysis of 14 available RCTs (all conducted in China) did not find that tiapride is significantly better than placebo [6], which is in contrast to the recommendations of the AAN [3] and both Chinese [11, 12] guidelines based on RCTs. Interestingly, a recent study on therapeutic drug monitoring in 49 pediatric patients (83.7% male, mean age = 12.5 years) found a positive correlation between tiapride dose (median 6.9 mg/kg, range 0.97–19.35) and serum concentration albeit with marked inter-individual variability. The variation in dose explained 57% of the inter-patient variability in tiapride serum concentrations; age, sex, and concomitant medication did not contribute to the variability. Tics improved in 83.3% of the patients. 27.1% of the patients had mild or moderate adverse effects [147].

A meta-analysis comparing the effects of different antipsychotics in TS [6] demonstrated that the most common adverse effects in patients with TS treated with tiapride are dizziness, nausea, and dry mouth, while EPS are rare [138, 145]. However, quite rarely EPS might be observed in case of a steep drug increase in the initial phase of treatment or with irregular drug intake. Of note, tiapride can be successfully used to treat (tardive) dyskinesias due to antipsychotics [145, 148].

### Noradrenergic agents

Noradrenergic agents such as clonidine and guanfacine are more commonly used in children and adolescents than in adults and mainly in those patients with a combination of ADHD and mild tics given their efficacy in treating ADHD symptoms in addition to tics.

#### Clonidine

For the treatment of tics, clonidine, an α-2 adrenergic agonist, has been used more commonly in America than in Europe [111] and is available as an oral and transdermal preparation. A systematic review [8] concluded that the balance of clinical benefits to harm favors the α-2 adrenergic receptor agonists clonidine and guanfacine (based on four studies with low risk of bias dating before 2011). However, the authors reported substantial heterogeneity with studies with transdermal application of clonidine being less effective compared to oral administration. The most recent meta-analysis of clonidine as treatment for TS pointed to a small standardized mean difference compared with placebo of 0.29 (95% CI [0.12–0.47]) [90]. This meta-analysis [90] indicated that the effect size of α-2 adrenergic agonist on tic reduction is much larger in children with tics plus ADHD (95% CI: 0.36–1.01) than in individuals with tics without ADHD (95% CI: − 0.06–0.36). Moreover, a prospective, open trial in 41 children and adolescents in whom previous treatment with a D2-dopamine receptor antagonist was ineffective or not well tolerated indicated a response rate of 63% after 12 weeks of treatment with a clonidine transdermal patch [149]. Unfortunately, the authors did not report effects on co-existing ADHD.

A systematic review of adverse effects of α-2 adrenergic agonists in children and adolescents with ADHD demonstrated hypotension, bradycardia, and sedation with clonidine as well as guanfacine [150]. Abrupt withdrawal of α-2 adrenergic agonists may cause rebound hypertension [151]. Therefore, blood pressure and pulse should be measured at baseline and monitored during dose adjustments and follow-up. In addition, monitoring of symptoms suggestive of cardiovascular problems (e.g., exercise intolerance, dizziness, and syncope) is recommended [152].

#### Guanfacine

Guanfacine, another α-2 adrenergic agonist, may reduce tics and improve ADHD symptoms in children and adolescents. However, in a recently published small-scale randomized double-blind placebo-controlled trial in children and adolescents (50% of the guanfacine, 22% of the placebo group suffered from co-existing ADHD) [153], guanfacine was not more efficacious than placebo in reducing tics. Previously, a meta-analysis of guanfacine as treatment for TS pointed to a standardized mean difference compared with placebo of 0.54 (95% CI [0.06–1.14]) [90].

The most common adverse effects of guanfacine are sedation, headache, fatigue, dizziness, irritability, upper abdominal pain, and nausea, with sedation and fatigue usually emerging within the first 2 weeks of dosing and then generally remitting [154]. Guanfacine may induce mania in children with a history or family history of bipolar disorder [155, 156]. Especially the extended release formulation of guanfacine may induce QTc prolongation [150], and therefore, patients should be monitored accordingly [3].

## Other agents

### Cannabis-based medicines

First reports of successful self-medication with the exocannabinoid cannabis date back to 1988 [157]. During the last years, more and more, mostly adult patients use cannabis as a self-medication and report beneficial effects [86, 158]. Indeed, there is an increasing number of case reports and small studies suggesting that cannabis-based medicines including cannabis flowers, cannabis extracts, and pure THC (dronabinol) might be effective in the treatment of tics and co-existing symptoms including ADHD. Since 2011, no new RCTs have been published. A recent meta-analysis on the two available small-scale RCTs (combined *n* = 41) demonstrated no significant benefit of THC compared to placebo as treatment of TS [159]. No serious adverse reactions were reported either, with only mild adverse reactions including dizziness, tiredness, and dry mouth [160, 161].

### Botulinum toxin

In addition to the use of pharmacological agents with systemic effects, there is some evidence for the efficacy of botulinum toxin injections to treat persistent well-localized motor and, sometimes, vocal tics by temporarily weakening the associated muscles, through the inhibition of acetylcholine release from peripheral motor nerve terminals. In European practice this approach is limited to older adolescents and adults in patients with insufficient response to other treatments. According to the AAN guidelines on TS [3] botulinum toxin as local application is probably more likely than placebo to reduce tics. This judgement as well as several reviews after 2011 on botulinum toxin in TS [74–85] are based on the only published randomized crossover trial of botulinum toxin injection versus placebo for the treatment of simple motor tics from 2001 conducted in 20 adolescents and adults [162]. Adverse reactions associated with botulinum toxin may include temporary soreness and mild muscle weakness including hypophonia when used in the throat region to treat disturbing vocal tics [163].

### Topiramate

Topiramate is a sulfamate modified fructose diacetonide with unknown mechanisms of action. There have been no new English-language RCTs, since a 12-week randomized controlled trial of topiramate versus placebo published in 2010 that showed superior effects of topiramate compared to placebo in 29 children and adults with TS [164]. However, the authors of a recent meta-analysis [96] summarizing a total of 15 studies from China involving 1070 participants aged 2–17 years concluded that topiramate is a promising medication with good efficacy and tolerability for children with TS compared to haloperidol and tiapride.

While generally well tolerated at low doses (25–150 mg/day) it may cause a variety of adverse effects, including cognitive and language problems, aggression or mood swings, paresthesia, nausea, sweating problems, and decreased appetite [165].

## Pharmacological treatment of tics in the context of co-existing psychiatric conditions

People with TS often suffer from co-existing problems, such as ADHD, OCD, mood disorders, anxiety, oppositional defiant disorder, and impulse control disorders (see Part I of this issue). The distress and burden associated with these co-existing conditions is often more significant to patients [166, 167] than the tics themselves. Although data are still limited [168], below, we present a possible approach for the treatment of co-existing psychiatric conditions in patients with TS.

### Attention-deficit/hyperactivity disorder (ADHD)

ADHD is prevalent in 30–50% of referred children with TS and is strongly associated with functional impairment [166, 167]. ADHD symptoms typically improve in adolescence [169], but some adults with TS may still need continued treatment for this co-existing disorder [170]. Several pharmacological trials have assessed medication for co-existing ADHD in TS. Across studies, therapeutic doses of methylphenidate, dextroamphetamine, clonidine, guanfacine, and atomoxetine reduce ADHD symptoms as well as tics in patients with TS, probably through allowing a better self-regulatory control [99]. The α-2 agonists clonidine and guanfacine are among the agents with the most favorable efficacy-versus-adverse events ratio but effect sizes vary [8, 9, 90]. While earlier studies described that stimulants exacerbated tics or even caused first tics in some individuals [171], more recent studies demonstrated that tics do not emerge or worsen under the treatment with short-acting [172, 173] or short- and long-acting stimulants [174]; however, a transient increase may occur. On the contrary, a mild reduction of tics may occur in the treatment with methylphenidate in children with tics plus ADHD [99]. In rare cases with a persistent increase of tics after introducing a stimulant, the use of atomoxetine may be a viable alternative, which may in general have a positive effect on tics via a reduction of ADHD symptoms [3, 103]. When treating with psychostimulants, some adverse events should be taken into consideration: sleeplessness, nervousness, headache, blood pressure raise, loss of appetite, weight loss, and gastro-intestinal complaints. According to an open-label study aripiprazole results in an effective reduction of tics, but affects ADHD symptoms only moderately [175].

### Obsessive–compulsive disorder (OCD)

Obsessive–compulsive behaviors are very common in people with TS, presenting frequently sensory-motor phenomena, such as urges and just-right feelings that may overlap with tics. Diagnostic criteria for OCD are met in up to 50% of people with tics [105, 176]. Trial data for OCD treatment in children (POTS II) suggest that individuals with tics respond as well to selective serotonin reuptake inhibitors (SSRIs) as those without tics and respond equally well to cognitive behavioral interventions [177] in contrast to an earlier study indicating a less favorable response to sertraline [178]. In addition, for OCD co-existing with TS, behavioral therapy approaches are the first-line treatment [3]. Small observational studies suggest that individuals without sufficient treatment response to behavioral therapy alone may benefit from an added SSRI [105]. Some fixed-dose trials of SSRIs showed that in the treatment of OCD higher doses are significantly superior to lower ones; there is, however, an expected greater adverse effect burden with higher doses of SSRIs [179]. It is worth noting that SSRIs may not only reduce OCD symptoms but also alter overall affect, anxiety, and stress sensitivity, which may lead to better self-regulation and tic suppression. However, this has not been documented in an RCT.

In treatment resistant OCD in the context of TS, antipsychotic augmentation of treatment with SSRI using aripiprazole and risperidone may be considered [180]; the subgroup of patients with OCD and co-existing tics had a particularly beneficial response to treatment with antipsychotic augmentation in a meta-analysis [179]. However, it is important to keep in mind the limited evidence base and the need for drug safety monitoring, as pointed out in an observational study including children with tic-related OCD [180] as well as a meta-analysis including adults with OCD (without tics) [181].

### Other co-existing psychiatric conditions

In addition to ADHD and OCD, people with TS are at risk of developing depression, anxiety disorders, oppositional defiant disorder, rage attacks, and mood disorders [176]. Co-existing mood disorders are more often seen in adolescents and adults than in children and in those with greater tic severity [3]. It is worth noting that there is an increased risk of suicidal ideation, suicide attempts, and suicide in people with TS, also when statistically controlling for other co-existing psychiatric conditions [182]. Unfortunately, there are no treatment studies to guide the clinician in treating these co-existing problems.

Guanfacine and clonidine can be effective in individuals with co-existing impulse control disorder [90]. Aripiprazole and risperidone are useful for co-existing irritability and aggressive behaviors [183–186].

Tics and stereotyped movements are frequent in Autism Spectrum Disorder, and a clear diagnostic distinction between them may be challenging to establish [28, 106]. Treatment with risperidone or fluoxetine may be considered in cases with stereotypies that are debilitating and involving harm and injury to self and others [187].

## Clinical recommendations for the pharmacological treatment of TS

Decisions about treatment of TS should be based on a thorough and broad diagnostic process (see Part I of this issue). Behavioral therapy approaches are recommended as first line treatment, based on assumed better tolerability of behavioral therapy, because behavioral approaches might strengthen the patients’ self-regulatory control [3, 188] (see Part V of this issue). However, these are not always locally accessible (a major factor in many countries) or feasible because of low introspective ability in young age or low IQ, or due to low motivation or ability to invest time and effort required for practicing in behavioral therapy. For individuals with clear impairments associated with their tics or with a preference for pharmacotherapy, after psychoeducation pharmacologic interventions may be considered alone or in addition to behavioral therapy. This concerns especially situations, where tics impair quality of life and cause subjective discomfort (e.g., pain or injury) or when tics result in sustained social problems (e.g., social isolation or bullying) or cause functional interference (e.g., impairment of academic achievements) [111]. In addition, pharmacological treatment acts faster, because prescription, dispensing, and intake of first dose are easier than planning and commencing behavioral therapy. Moreover, first treatment effects are often seen within a few days, while after behavioral therapy first beneficial effects in most cases cannot be observed until after a few weeks. Therefore, pharmacotherapy may be preferred in situations, where a rapid tic reduction is urgently required.

The waxing and waning course (including its time course) of the tics in each individual should be taken into account when deciding on starting therapy and when evaluating treatment effects.

Independently from the individual factors that result in the decision to start pharmacological treatment of tics it is important to inform patients and their parents about what can be achieved by this kind of treatment to avoid too high expectations. On average, a tic reduction of 50% can be expected. However, some patients report a reduction of 90%, while others feel no or only minimal improvement.

The decision to propose a treatment with a specific agent is an individual choice made by the clinician, in collaboration with the patient and family and depends on the patient’s needs, preferences, and priorities as well as on the physician’s preferences, experience, and local regulatory requirements.

During the last decades, several agents have been suggested and used as rational medication for the treatment of tics. Based on evidence from RCTs and on clinical experience aripiprazole, tiapride, and risperidone for TS as well as clonidine and guanfacine for TS and co-existing ADHD are the best established options, all on the basis of off-label use. In general, we recommend a “start low, go slow” drug up-titration, meaning that the therapy should be initiated with the lowest dose possible and gradually increased. It is important to bear in mind that the antipsychotic dosages normally used for the treatment of tics are considerably lower than those used to treat psychotic disorders.

Depending on its individual receptor binding profile, each agent bears the risk of specific adverse effects. Therefore, not only effectiveness but also potential adverse effects of each agent should be taken into consideration when deciding about the most suitable agent for a patient with TS. Most pharmacological treatments discussed in these guidelines have well known adverse effects, including weight gain, drug-induced movement disorders, elevated prolactin levels, sedation, and effects on heart rate, blood pressure, and electrocardiograms. Therefore, careful monitoring of adverse events is recommended (see Table 2). In case of treatment discontinuation, gradual tapering off antipsychotic medications is recommended to avoid withdrawal dyskinesias [3].

**Table 2:** Most common medications for Tourette syndrome and other chronic tic disorders

Medication	Indication	Start dosage (mg)	Therapeutic range per day (mg)	Effect size*	Confidence in the quality of the evidence**	Very common adverse events (> 10%)***	Physical and laboratory Examinations at the start and at follow-ups
α-2 adrenergic agonists							
Clonidine	ADHD/TS	0.025	0.025–0.3 (titrated according to BP and HR)	0.29 (0.12–0.47) [90]	Moderate	Dizziness, orthostatic hypotension, dry mouth	Blood pressure, ECG
First generation antipsychotics							
Haloperidol	TS	0.25–0.5	0.25–3.0	3.20 (0.14–6.52) [6]	Moderate	Agitation, insomnia, EPS, hyperkinesia, headache	ECG, weight
Pimozide	TS	0.5–1.0	1.0–4.0	0.42 (−0.07–0.90) [6]	Low	Dizziness, somnolence, hyperhidrosis, nocturia	ECG, weight
Newer antipsychotics							
Aripiprazole	TS	2.50	2.5–30	4.74 (1.06–8.67) [6]	Moderate	Somnolence, sedation	Weight, blood lipids, and glucose
Risperidone	TS/DBD	0.25	0.25–3.0	3.47 (0.37–6.87) [6]	Moderate	Insomnia, sedation/somnolence, parkinsonism, headache	Weight, prolactin, blood lipids, and glucose
Benzamides							
Tiapride	TS	50–100 (2 mg/kg)	100–600 (2–10 mg/kg)	0.47 (−3.89–5.06) [6]	Moderate	Hyperprolactinemia*, sleepiness, insomnia, agitation, impassivity, vertigo, headache	ECG, weight, prolactin
Others							
Botulinum toxin	TS	Vocal tics: 1–2.5 U Motor tics: 50–75 U	1–2.5 75–250	1.27 (0.51–2.03) [4]	Moderate	Weakness of the injected muscles	

*DBD* disruptive behavior disorder; *OCB* obsessive–compulsive behavior; *TS* Tourette syndrome; *ADHD* attention-deficit/hyperactivity disorder; *BMI* body mass index; *EP*S extrapyramidal symptoms; *BP* Blood pressure; *HR* heart rate; *ECG* electrocardiogram

Information on the adverse effects stems from the official Summaries of product characteristics, if no very common Adverse Events (> 10%), *standardized mean difference compared with placebo (including 95% confidence interval; positive number pointing to efficacy) according to the most recent meta-analysis (for botulinum toxin based on a single study), as referenced ** based on the Grading of Recommendations, Assessment, Development and Evaluations (GRADE) as reported by AAN [4] ***common Adverse Events (< 10% and > 1%) are provided

An important aspect when choosing an agent for a patient is also the presence of co-existing conditions. Often, the co-existence of ADHD or OCD, as well as mood, anxiety, or impulse control disorders may be more disturbing to the patient than the tics [167] and may thus have important implications for the choice of medication. Evidence for those choices is still limited, but this differentiation already presents an important step towards an individualized approach to medication in TS.

While the evidence-based practice recommendations of the AAN did not present a hierarchical recommendation what agent should be given first, the ESSTS survey indicates that aripiprazole is now the most commonly used agent for the pharmacological treatment of TS for both age groups (children and adolescents, adults). This may be the result of several factors, one being the unique pharmacological profile as a dopamine partial agonist [189], but also the availability of several RCTs with sufficient sample sizes that document a favorable benefit-risk ratio, predominantly being the result of its positive profile of adverse effects [69, 70, 72]. Positron emission tomography studies demonstrated that the clinical effect of an antipsychotic emerges when more than 65% of striatal dopamine D2 receptors are blocked, and EPSs become apparent when the receptor blockade exceeds 80% [190]. Thus, in the ideal antipsychotic therapy (antipsychotic efficacy without EPSs), about 70% of striatal dopamine D2 receptors are blocked. When tight antipsychotics bind 70% of D2 receptors, the remaining 30% are available for endogenous dopamine to bind. This means that dopaminergic transmission is reduced to 30%, and both tonic/phasic components are suppressed equally. In one study, aripiprazole was effective at a dose of up to 20 mg, where 10% or fewer D2 receptors were available for endogenous dopamine to bind; however, EPSs did not appear, because aripiprazole exerted a partial dopaminergic agonistic activity [191].

Tiapride, the second most commonly prescribed agent in children and adolescents with tics, especially in Germany, has a similar working mechanism as aripiprazole, showing a maximum of 80% of dopamine receptor occupation even in the presence of excess tiapride concentrations [145]. Interestingly, for two of the most commonly used agents according to the ESSTS survey there is evidence from pharmacodynamic studies explaining their low (aripiprazole) or very low (tiapride) potential for EPS compared to haloperidol [69, 70, 138, 145].

Another recommended antipsychotic agent, risperidone, actually has a good evidence base, but is associated with weight gain and metabolic adverse effects.

The European survey documented that noradrenergic agents are the third most given agents regarding both age groups together. Importantly, noradrenergic agents have a low effectiveness in patients with tics only, but this substantially increases in patients (particularly children and adolescents) with the combination of tics and ADHD, both for reducing tics and symptoms of ADHD [8, 90]. Therefore, we recommend noradrenergic agents as first line treatment of mild to moderate tics in patients with co-existing ADHD, but less in those without co-existing ADHD as there they have only minimal benefits. However, in some patients with mild tics only, noradrenergic agents may be more acceptable than antipsychotics, based on more favorable adverse effects.

In treatment resistant cases, treatment with agents with sometimes a still limited evidence base and less frequently prescribed by ESSTS experts might be considered. Reasonable choices include antipsychotics including haloperidol, pimozide, quetiapine, sulpiride, and amisulpiride as well as cannabis-based medicines, topiramate, and botulinum toxin injections.

Haloperidol is still relatively often used in adults with TS, but rarely mentioned by any ESSTS expert as treatment option for children and adolescents. Its declined use can be explained by its unfavorable adverse effect profile compared to other antipsychotics, even though haloperidol is the only officially licensed substance for TS and tics in Europe, and has a long tradition and established efficacy in the treatment of TS, with relatively low costs.

## Current limitations and future directions

In the light of the limited existing evidence several questions remain unanswered: most importantly, the effectiveness of combinations of behavioral therapy with pharmacological treatment and of different agents needs further trials. Studies directly comparing different agents or combinations of agents in TS are rare, and there is currently only one study [192] available that compared pharmacological treatment with behavior therapy, yielding equal effects within a study period of 10 weeks. Moreover, the study periods of published trials on pharmacological treatment of TS were quite short, e.g., in view of the natural waxing and waning course of tics in TS. In addition, research should be conducted on treatment sequencing and decision-making and for whom particular sequences of treatment are most effective [3]. Another area in need of further evidence is the treatment of patients with co-existing conditions. Moreover, questions around how to deal with treatment refractoriness remain unanswered [193]. The risk of adverse events when using specific agents needs further exploration, e.g., sudden death due to QTc prolongation [116], hyperprolactinemia and its consequences [109], and weight gain [128]. In addition, the questions of optimal treatment duration, as well as long-term outcome after discontinuation of a pharmacological treatment of tics remain unanswered. These important points for the pharmacotherapy of TS are still open to discussion due to a non-existent or too small base of evidence and are important areas for future research. Unfortunately, the number of new agents that might be effective as treatment of TS is limited. Perhaps most promising are the Chinese herbal medicine products 5-ling granule and Ningdong granule, which were classified as compounds showing moderate confidence in evidence of treatment effects according to the AAN guidelines, based on well-powered RCTs conducted in China. However, these products are currently not available to clinicians on the European market. One final future step to improve pharmacological treatment of TS would be precision medicine as well as personalized medicine [194] by prior genetic testing or the use of other neurobiological markers [195]. This approach, however, is still an aspiration for neuropsychiatric disorders, such as TS.

## Footnotes

1 We use the term TS in these guidelines, wherever information also applies to other forms of tic disorders. Only if there are substantial, well-known differences between TS and other forms of tic disorders we use TS or other terms, e.g. transient or chronic motor tic disorder.
